# IPEC-J2 as a cellular model for studying intestinal mucus

**DOI:** 10.1038/s41598-025-32027-5

**Published:** 2025-12-09

**Authors:** Frédéric Dessauge, Annie Vincent, Christine Trefeu, Frédérique Mayeur-Nickel, Sandra Wiart-Letort, Adam Macierzanka, Martin Beaumont, Myriam M.-L. Grundy

**Affiliations:** 1 PEGASE, INRAE, Institut Agro, Saint Gilles, 35590 France; 2 Faculty of Chemistry, Gdańsk University of Technology, Gabriela Narutowicza 11/12, Gdańsk, 80-322 Poland; 3 GenPhySE, Université de Toulouse, INRAE, ENVT, Castanet‑Tolosan, 31326 Toulouse, France

**Keywords:** Biochemistry, Biological techniques, Cell biology, Gastroenterology

## Abstract

Mucus in the proximal small intestine serves critical protective and transport functions, regulating nutrient absorption to enterocytes. The porcine jejunal epithelial cell line IPEC-J2 is widely used to study epithelial barrier function, yet its capacity to express mucins remains inconsistently described. This study aimed to investigate the ability of IPEC-J2 cells to express mucins under various culture conditions: 5% or 10% porcine serum (5PS, 10PS), with agitation (5PSAg and 10PSAg) and air-liquid interface (ALI). Mucus production was assessed using functional and structural approaches. Expression of secreted mucin MUC2 and goblet cell marker TFF3 was limited under most conditions but markedly enhanced in ALI and 5PSAg cultures. Immunohistochemistry revealed membrane-associated mucins (MUC3, MUC13) although MUC13 localisation differed between IPEC-J2 and jejunal tissue. Flow cytometry revealed that ~ 8% of IPEC-J2 cells expressed MUC2 in 5PSAg, comparable to the proportion of jejunum’s goblet cells in vivo (~ 5%). This study demonstrated that IPEC-J2 cells can differentiate into mucus-secreting cells under specific culture conditions, and they are a suitable in vitro model for investigating interactions between mucus and food components, providing a valuable tool for nutritional research.

**Supplementary Information:**

The online version contains supplementary material available at 10.1038/s41598-025-32027-5.

## Introduction

The mucosa of the gastrointestinal tract (GIT) is lined by a range of epithelial cells covered by a layer of mucus. In the small intestine, the epithelial cells are organised into a single layer that consists of absorptive cells (enterocytes), goblet cells (secrete mucus), enteroendocrine cells (secrete hormones), Paneth cells (produce antimicrobial peptides and growth factors), undifferentiated cells (pluripotent stem cells located at the base of the crypts of Lieberkühn), as well as less characterised cells - the microfold cells (M cells), BEST4 + cells, and tuft cells^1,2^. Between the cells and the mucus layer lies the glycocalyx, a vast network of glycoproteins (glycosylated proteins and lipids) bound to the epithelial cells^3^. The intestinal mucus is composed primarily of water (< 83%), mucins (up to 5%), proteins (6%) and lipids (4%)^4^, with extracellular DNA also contributing to its composition in the small intestine, where it plays an important structural role^5^. Mucins (MUC) are glycoproteins composed of a core protein, rich in proline, threonine and serine (called the PTS region), to which glycan side chains (O-linked oligosaccharides) are attached. Those are large molecules with a size that has been estimated to range from 0.2 to 10 MDa; the protein core can contain more than 5 000 amino acids and the glycan side chains occupy at least 70% of its molecular weight^6,7^.

Different intestinal epithelial cell types contribute to mucin production. Goblet cells secrete gel-forming mucins (e.g., MUC2 and MUC5AC), which confer viscoelastic properties to the mucus layer^8^. In contrast, enterocytes express membrane-bound mucins (MUC3, MUC13, MUC17) that constitute the glycocalyx^9,10^. These membrane-bound mucins differ structurally and functionally from the gel-forming mucins produced by goblet cells. The mucus layer acts as a semipermeable barrier that prevents bacteria and pathogens from being in contact with the underlying epithelium; while facilitating the transport of the digesta along the GIT as well as allowing the passage of nutrients and other small molecules that are able to diffuse through the mucus pores and channels towards the epithelial cells^11^. Depending on its location within the GIT, it is generally accepted that this mucus can be formed of a single layer (loosely adherent mucus outer layer in the small intestine and colon) or a double layer with the additional presence of the inner firmly adherent mucus layer (predominantly in colon)^3,7,12^. In this work, we will focus on the small intestine, and more specifically the jejunum, since the IPEC-J2 (jejunum porcine cell line) used in this study is a model of the jejunum, which is the main site of absorption of nutrients, such as amino acids and peptides^13^.

IPEC-J2 is used as a porcine model to study epithelial barrier function, the transport of molecules (absorption) through the intestinal cells, and the interactions of these cells with compounds present in the lumen (digesta)^14,15^. This epithelial cell line was isolated from neonatal piglet mid-jejunum in 1989^16^. Their mucus secretion capabilities remain inconsistently reported in the literature, with limited characterisation of their differentiation into mucus-producing goblet cells^17–21^. Questions therefore remain as to whether or not mucins are secreted by the IPEC-J2 cells; and if so, by which type of cells, enterocytes or goblet cells? Furthermore, does the mucus layer exhibit some of the structural complexity of the mucus found in vivo?

In this work, our objective was to determine the suitability of IPEC-J2 as a physiologically relevant model for investigating mucus–food compounds interactions in the jejunum. We used morphological and functional complementary approaches to firstly describe the jejunum of piglets. Then, IPEC-J2 cells were comprehensively characterised under various culture conditions, to identify if they can be a good model of porcine jejunum, with a focus on mucus production.

## Results

### Mucin identification in piglet jejunal tissue

**Fig. 1:**
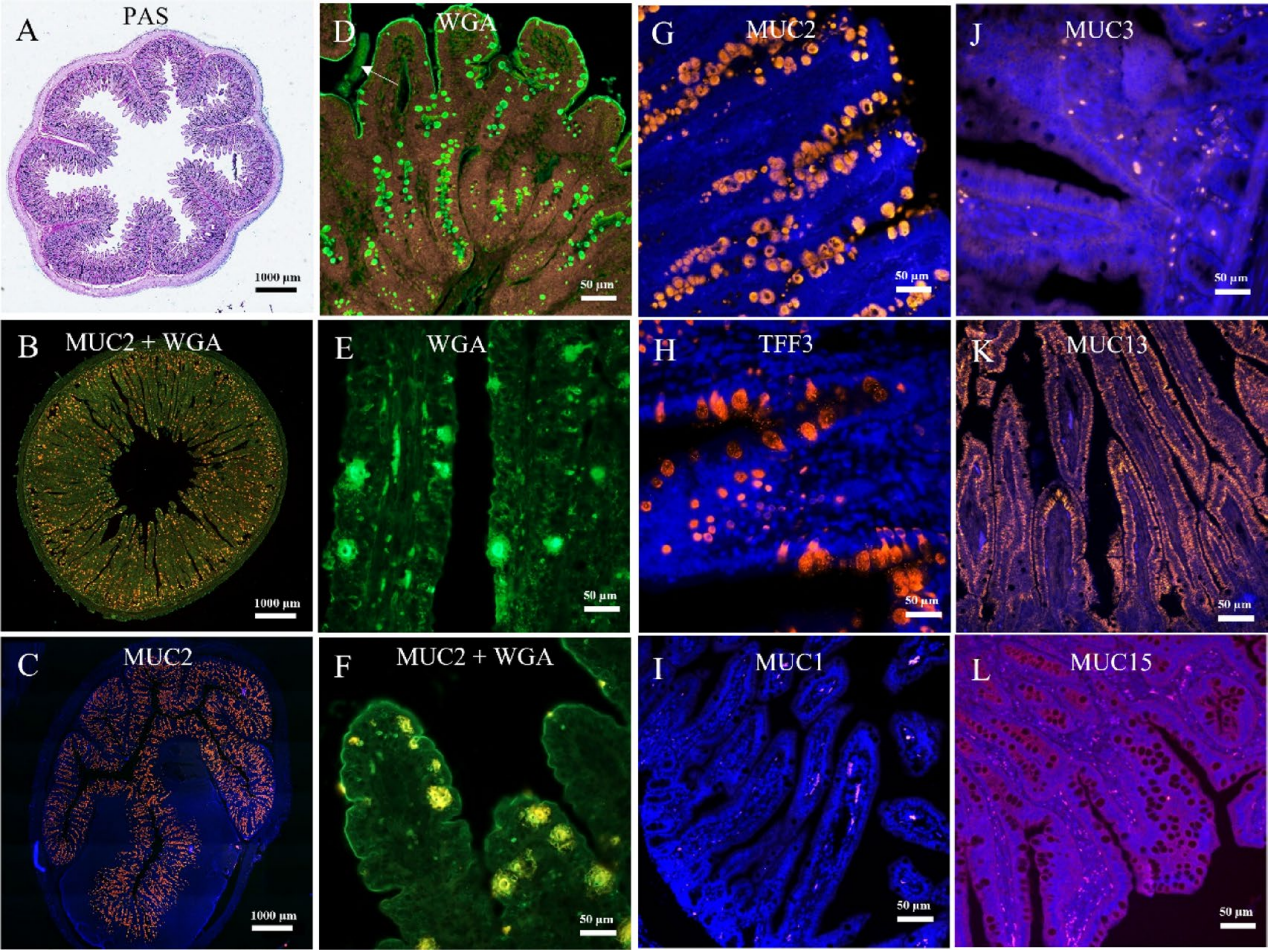
Representative images of jejunal tissue sections from piglets. PAS staining (**A**) or immunofluorescence of mucins (I, MUC1; **B**, **C**, **F** and **G** MUC2; **J**, MUC3; **K**, MUC13; and L, MUC15), and trefoil factor 3 (TFF3, H). Wheat germ agglutinin (WGA, **B**, **D**–**F**) lectin staining revealed surface and secreted glycoconjugates, including goblet cell mucus and enterocyte glycocalyx components. Nuclei were counterstained with DAPI (blue). Scale bars: 1000 μm–50 μm. Images are representative of three independent experiments (*n* = 6 piglets, for each piglet 3 sections of the jejunum were obtained).

Staining with PAS and WGA performed on the jejunum of piglets showed glycans distributed within the tissue, inside globular structures (goblet cells), and at the surface of the microvilli (Fig. 1A-E). PAS and WGA stains bound to glycoproteins and thereby enable the identification of both goblet cells and membrane-bound mucins. Furthermore, some secreted mucus remained despite the preparation of the sample that removed most the loosely adhered mucus layer (white arrow in Fig. 1D).

Labelling of the samples with MUC2 antibodies (Fig. 1C, F and G; orange fluorescence) confirmed that MUC2 was present within the goblet cells. In Fig. 1H, Trefoil Factor 3 (TFF3; orange fluorescence) was used as a specific marker of goblet cells. The size of the cells and localisation within the tissue were consistent with the images obtained with PAS and WGA staining, although the cells seemed less numerous.

MUC3 (orange fluorescence, Fig. 1J) was detected but to a lesser extent than MUC13 (orange fluorescence, Fig. 1K). MUC13 labelling appeared at the surface of the enterocytes, whereas MUC3 was present only in a few locations within the villi. MUC1 (Fig. 1I) was not detected in the jejunal tissue, which was expected as it is mainly present in the colon, according to the studies performed in humans^22^. The same was observed for MUC15 (Fig. 1L).

**Fig. 2:**
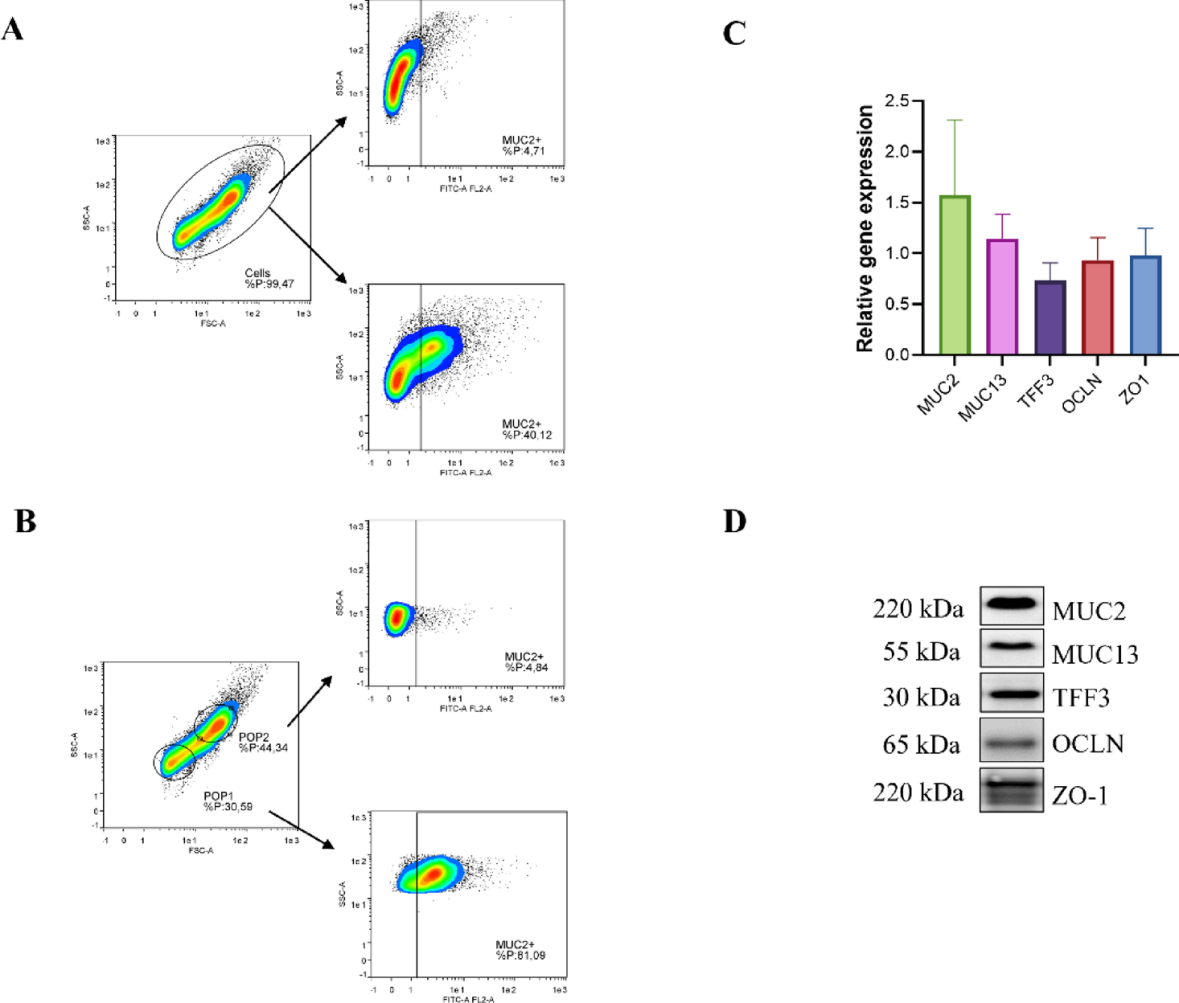
Protein and gene expression in jejunal tissue from piglets. Flow cytometry analysis of trypsin-dissociated jejunal cells stained with anti-MUC2-FITC antibody reveals MUC2 + goblet cell population (~ 5% of total cells). Gating thresholds for each isotype control are indicated by solid lines (**A**). Scatter plot analysis distinguishing two cell populations based on forward scatter (FSC) and side scatter (SSC) parameters, with MUC2 expression defining goblet vs. enterocyte phenotypes (**B**). Western blot analysis confirms protein expression: MUC2 (220 kDa), MUC13 (55 kDa), TFF3 (30 kDa), ZO-1 (220 kDa) and occludin (OCLN, 65 kDa) (**C**). Quantitative mRNA expression of barrier-forming genes (MUC2, MUC13, TFF3, OCLN, ZO-1) normalised to housekeeping genes YWHAZ, B2M, and RPL4 (**D**) Data are mean ± SEM of three biological replicates (*n* = 6, for each piglet three measurements were performed).

Flow cytometric analysis of MUC2 expression following jejunum cell dissociation revealed approximately 40% positive events within the total population (Fig. 2A). Further examination of the flow cytometry data identified two distinct populations (POP1 and POP2) distinguished by differences in size and morphological characteristics (Fig. 2B). Population 1 (POP1) demonstrated high MUC2 expression with over 80% positive signals, likely representing extracellular mucus fragments or cellular debris, whereas population 2 (POP2) exhibited 4.84% MUC2 expression, corresponding to goblet cells. RT-qPCR and Western blot analysis confirmed the immunochemistry results (Fig. 2C). The size of MUC2, presumably corresponding to the protein core (PTS region), was about 220 kDa (Fig. 2D). Occludin (OCLN, 65 kDa) and ZO-1 (220 kDa) were also detected.

### Kinetics characterisation of IPEC-J2

**Fig. 3:**
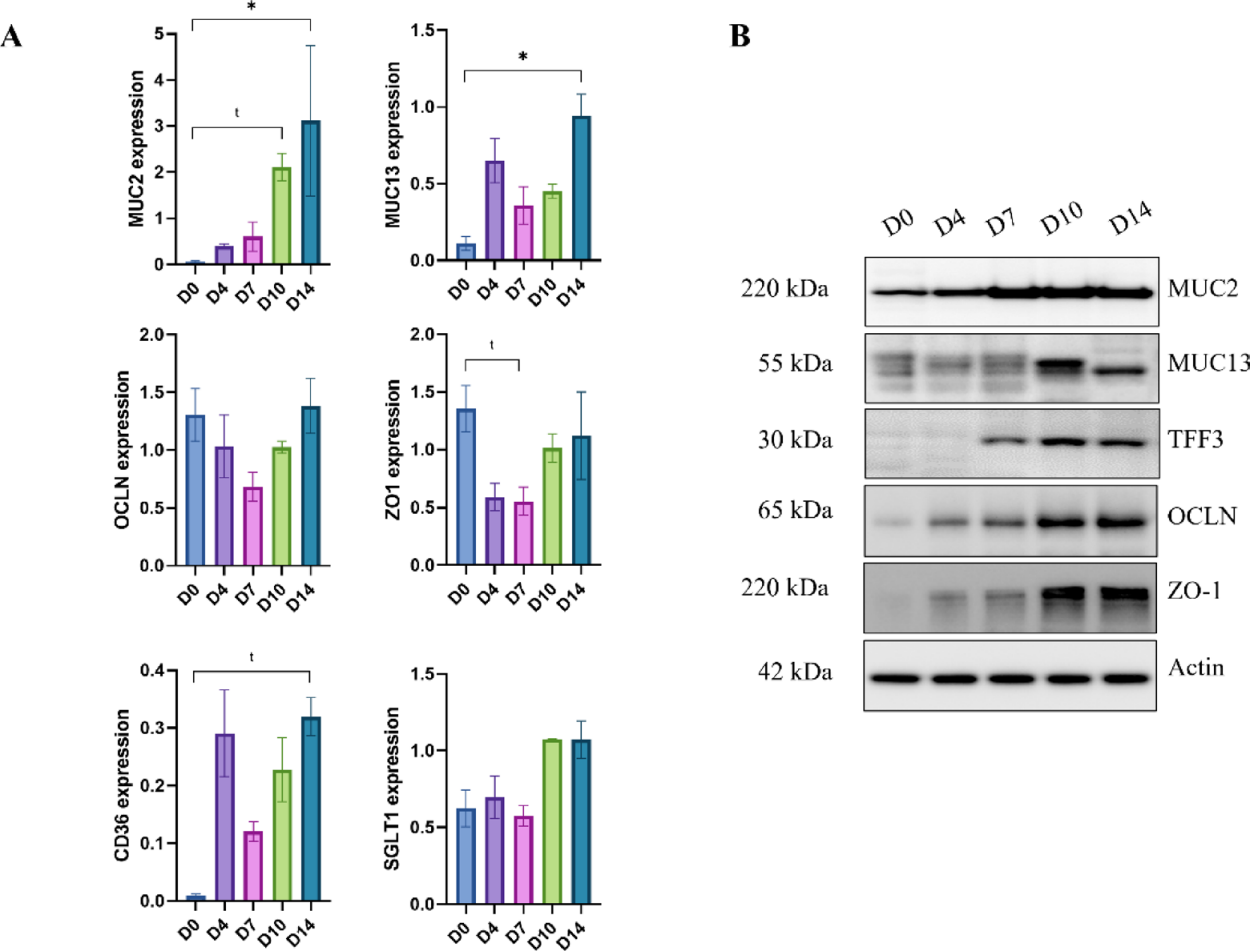
Temporal expression of mucins and tight junction protein in IPEC-J2 cells cultured under four conditions: 5% or 10% porcine serum with static (5PS or 10PS) or orbital agitation at 200 rpm (5PSAg or 10PSAg). Relative gene expression measured by qPCR (**A**). Note MUC2 peak expression at day 10–11, coinciding with goblet cell maturation. Representative Western blots demonstrating protein accumulation patterns for MUC2, MUC13, TFF3, ZO-1, OCLN, with actin as loading control (**B**). Data represent means ± SEM (*n* = 3). Statistical analysis: One-way ANOVA with Tukey’s post-hoc test; *P < 0.05 vs. day 0, *“t”* indicates statistical trend (0.05 < *P* < 0.10).

To establish the optimal culture duration for IPEC-J2 differentiation, we investigated the temporal expression patterns of both mucins and tight junction proteins over a 14-day period using the 5PS culture condition. This kinetic analysis was essential to determine when the cells achieve a mature phenotype resembling functional jejunal epithelium. RT-qPCR analysis revealed distinct temporal patterns in mucin expression during IPEC-J2 differentiation (Fig. 3A). MUC2 expression demonstrated a progressive increase throughout the culture, reaching maximum levels at day 14. This increase in MUC2 expression indicates a gradual differentiation of cells toward a mucus-secreting phenotype. The expression of TFF3, a goblet cell marker, closely paralleled that of MUC2 at the protein level, showing progressive increases until day 14 (Fig. 3B).

MUC13, a membrane-bound mucin that forms part of the glycocalyx, was also expressed throughout the differentiation (Fig. 3A). Western blot analysis provided additional insights into MUC13 protein dynamics (Fig. 3B). During the early stages of cell growth (days 0–7), multiple protein bands corresponding to different forms or processing states of MUC13 were detectable. However, by day 14, only a single predominant band remained, suggesting that the mucin had undergone complete maturation and processing.

The establishment of a functional epithelial barrier was monitored through the expression of tight junction proteins, occludin and ZO-1. Western blot analysis confirmed the presence of occludin at 65 kDa and ZO-1 at 220 kDa (Fig. 3B). Both proteins showed progressive increases in expression as the cells differentiated while mRNA expressions were not significantly different (Fig. 3A). The increasing expression of these tight junction proteins paralleled the mucin expression patterns, indicating coordinated development of both barrier integrity and mucus production capabilities. The upregulation of occludin and ZO-1 reflects the strengthening of cell-cell adhesion and the formation of a mature, polarized epithelial monolayer. In addition to mucins and tight junction proteins, the expression of nutrient transporters was monitored to assess overall enterocyte differentiation. Thus, CD36 (fatty acid translocase) and SGLT1 (sodium-glucose co-transporter 1) showed respectively slightly increased or stable expression throughout differentiation (Fig. 3A), indicating that IPEC-J2 cells early expressed and maintained their absorptive capacity alongside developing mucus-secreting functions.

Taken together, these kinetic data demonstrate that IPEC-J2 cells require a minimum culture period of 14 days to achieve full differentiation under 5PS conditions. These findings are consistent with previous reports demonstrating that extended culture periods are necessary to obtain IPEC-J2 monolayers with physiologically relevant barrier properties^23,24^.

### Mucin secretion under different culture conditions

The main objective of this work was to identify the conditions under which mucin secretion, particularly MUC2, was optimal and as close as possible to the one from native piglet jejunal tissue. To achieve this, we used 4 culture conditions: 5PS, 5PSAg, 10PS, and 10PSAg.

**Fig. 4:**
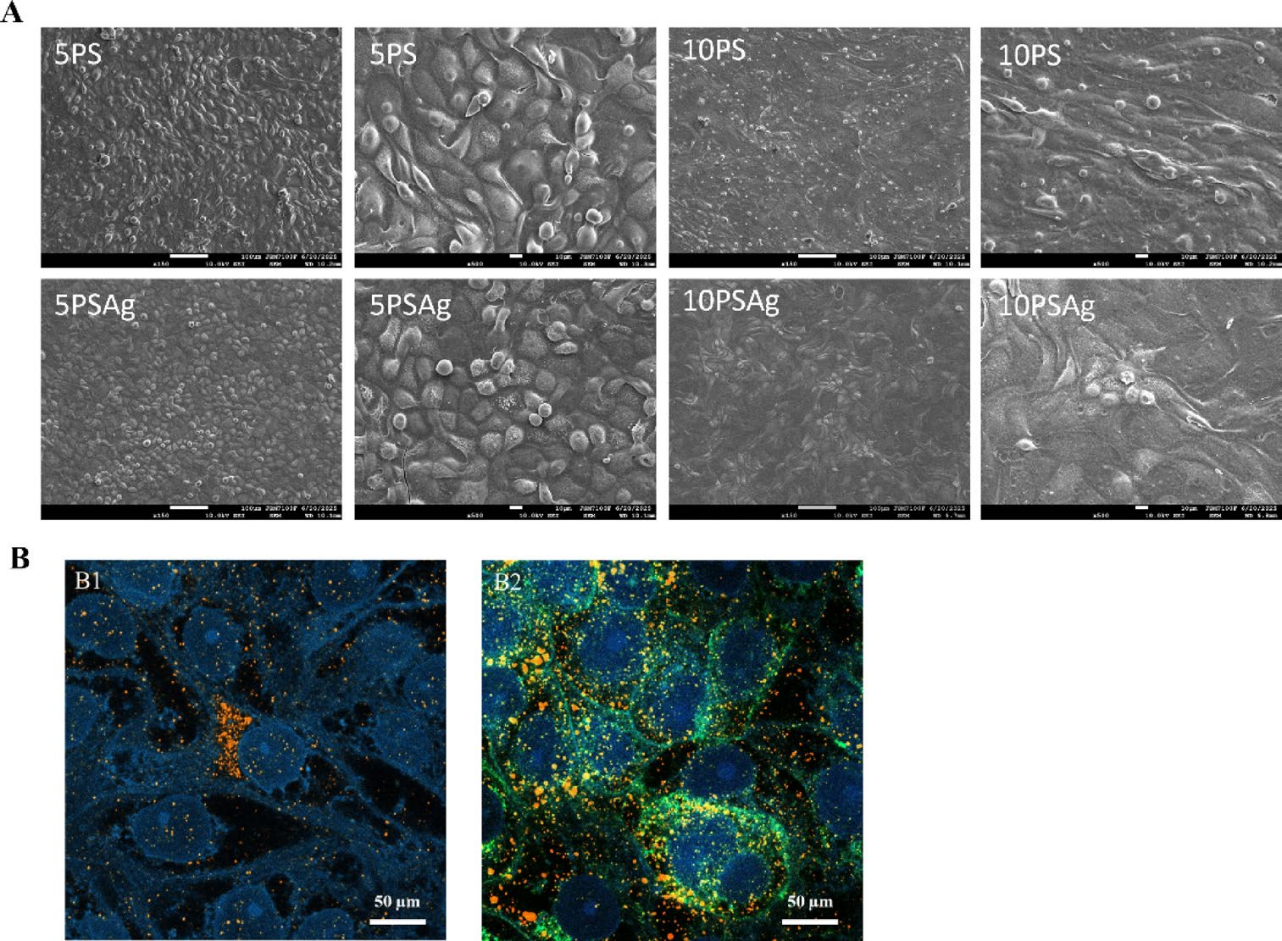
Electron and confocal microscopy of the IPEC-J2 cells cultured under four conditions: 5% or 10% porcine serum with static (5PS or 10PS) or orbital agitation at 200 rpm (5PSAg or 10PSAg). Scanning electron microscope illustrating the cells organisation (**A**). Confocal images of cells cultured under 5PSAg condition and labelled with MUC2 antibody (orange) and WGA (green) showing mucin secretion by goblet cells (**B**). Note the different shape of cells from which the MUC2 originate. The mucins spread over the monolayer. Scale bars: 50 μm.

Images obtained by light microscopy showed IPEC-J2 monolayer similar in appearance regardless of the culture conditions (Fig. S1); to be able to identify potential differences the cells were also observed under SEM (higher resolution). Using that technique, the monolayer appeared well defined with adherent cells, in particular for 5PS and 5PSAg (Fig. 4A). Apical microvilli were visible, validating the differentiation of the cells into enterocytes (Fig. S3). The cells cultured under 10PS and 10PSAg looked elongated, with their shape being more irregular than those cultured under 5PS. Confocal images of the 5PSAg condition show a cell from which MUC2 (labelled in red) is secreted (Fig. 4B), the other mucins are labelled in green (WGA).

**Fig. 5:**
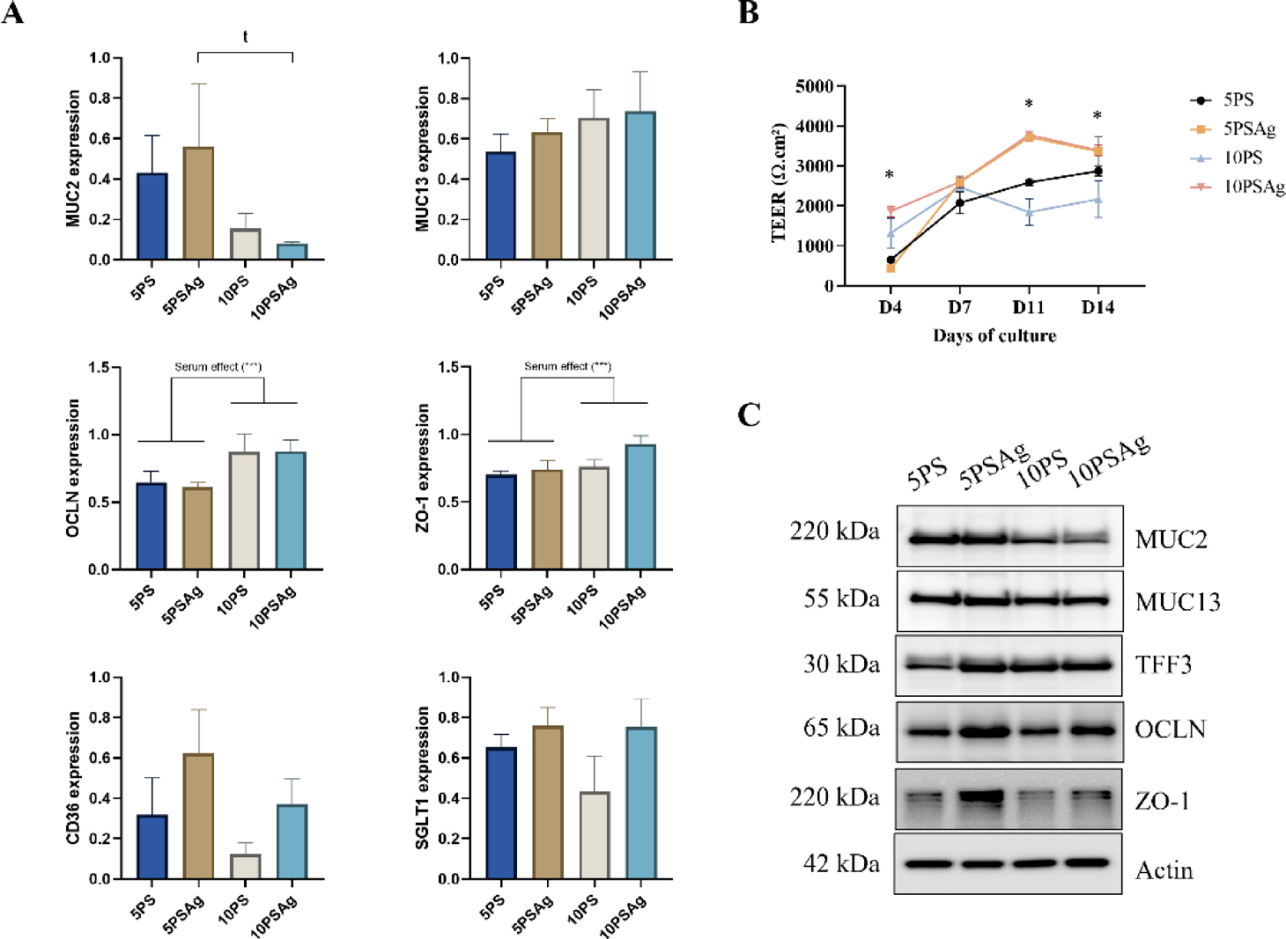
Functional studies of IPEC-J2 cells cultured under four conditions: 5% or 10% porcine serum with static (5PS or 10PS) or orbital agitation at 200 rpm (5PSAg or 10PSAg). Relative gene expression of barrier genes (OCLN, ZO-1), transporters (CD36, SGLT1) and mucins (MUC13, MUC2) measured by qPCR (**A**). Agitation enhanced mucin gene expression, particularly under 5% PS conditions. Transepithelial electrical resistance (TEER) measurements demonstrated enhanced barrier integrity under agitated conditions, with characteristic microvilli-associated resistance patterns peaking at day 11 (**B**). Western blot of protein expression (MUC2, MUC13, TFF3, OCLN, ZO-1) with actin as loading control showing optimal mucin production in 5PSAg conditions (**C**). Data represent means ± SEM. (*n* = 3). Statistical analysis: Two-way ANOVA with Tukey’s post-hoc test; *P < 0.05, *“t”* indicates statistical trend (0.05 < *P* < 0.10).

The TEER measurements at days 11 and 14 indicated significant differences between the cells cultured under agitation (5PSAg and 10PSAg) compared to those analyses under static conditions (5PS and 10PS) (Fig. 5B). Both conditions, 5PSAg and 10PSAg, had similar values at 14 days, that were lower compared to day 11, which is characteristic of microvilli development^25^. Occludin and ZO-1 mRNA expressions were significantly increased with the serum concentration (Fig. 5A). MUC2 expression was significantly higher for 5PSAg compared to the other conditions. This trend was also observed in Western blotting for MUC2 and TFF3 (Fig. 5C). Genes coding for nutrient transporters (CD36 for lipids, and SGLT1 for glucose) tended to be differentially expressed with the agitation (Fig. 5A).

**Fig. 6:**
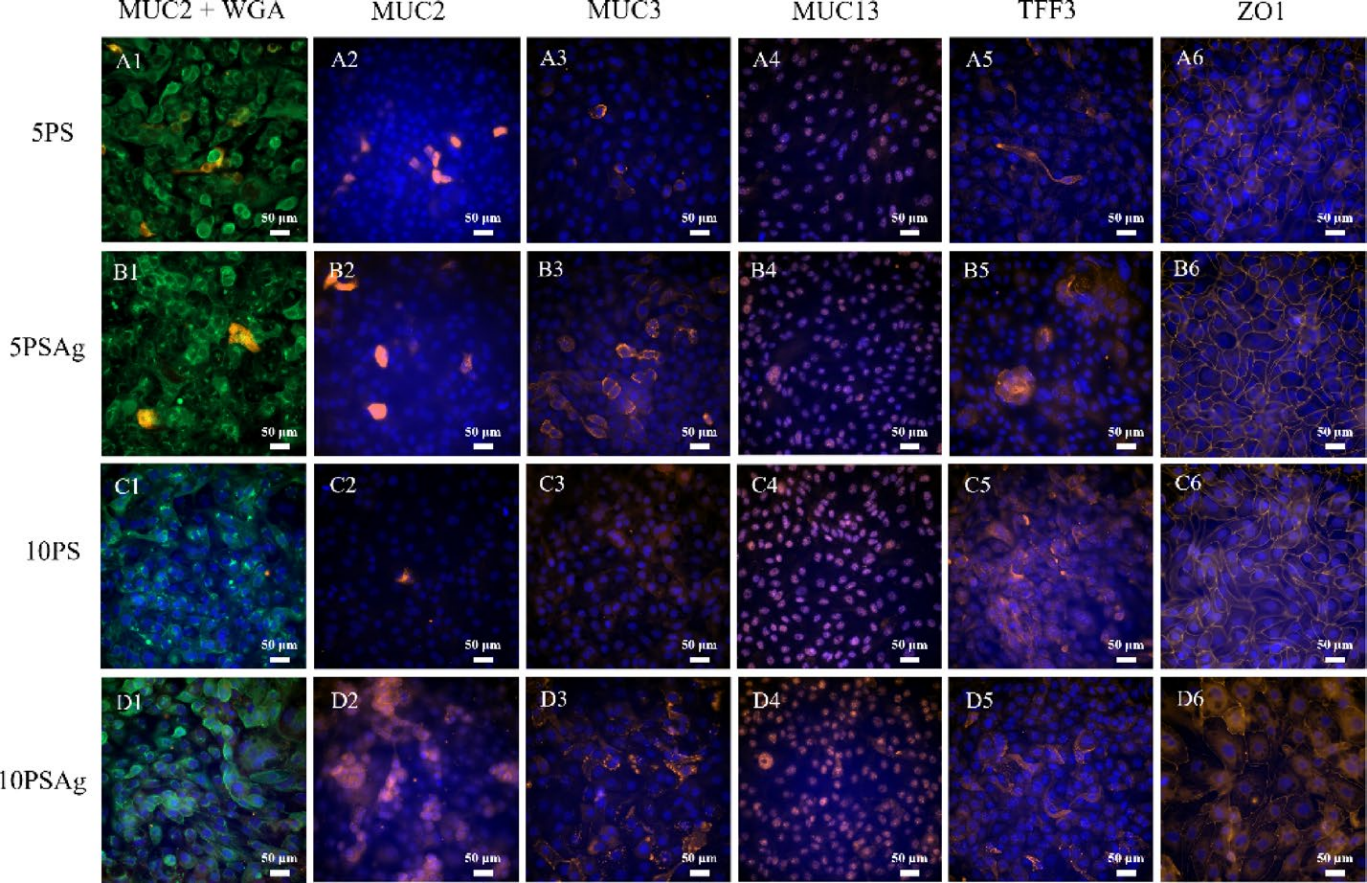
Representative immunofluorescence microscopy images of IPEC-J2 cells cultured with 5% or 10% porcine serum with static (5PS, **A**, or 10PS, **B**) or orbital agitation (5PSAg, **C**, or 10PSAg, **D**), showing subcellular distribution of mucins labelled with WGA and MUC2 (1), MUC2 (2), MUC3 (3), MUC13 (4), TFF3 (5) and tight junction protein ZO-1 (6). Dual MUC2 (orange) /WGA (green) staining demonstrates secreted mucin distribution from discrete goblet-like cells under 5PSAg conditions, most closely resembling native tissue patterns. MUC13 localises perinuclearly during synthesis/processing, while MUC3 shows membrane-associated distribution enhanced by mechanical stimulation. ZO-1 forms continuous intercellular junctions except under 10PSAg conditions showing irregular cell morphology. Nuclei were counterstained with DAPI (blue). Scale bars: 50 μm. Images representative of three independent experiments (*n* = 3).

Immunofluorescence staining for the tight junction protein ZO-1 revealed distinct organisational patterns across culture conditions. Under 5PS, 5PSAg, and 10PS conditions, ZO-1 was evenly distributed along cell-cell borders, forming continuous peripheral rings around individual cells that delineate the boundaries of the epithelial monolayer (Fig. 6A, B, and C). This regular ZO-1 distribution pattern is characteristic of well-differentiated, polarised epithelial cells with intact intercellular junctions. However, the 10PSAg condition displayed markedly aberrant ZO-1 organisation (Fig. 6D). The disrupted staining pattern correlated with pronounced morphological abnormalities observed in these cells. Specifically, DAPI nuclear staining revealed that many cells under 10PS and particularly 10PSAg conditions contained multiple nuclei, indicating either incomplete cytokinesis or cell fusion events (Fig. 6C6 and D6). Quantitative analysis of cell density from these images confirmed these morphological differences: 10PS cultures contained 105 cells per field and 10PSAg only 74 cells per field, compared to 203 cells for 5PS and 165 cells for 5PSAg. This significant reduction in cell number, combined with the presence of multinucleated cells, demonstrates that the 10PS conditions, especially with agitation, promote the formation of abnormally large, deformed, and potentially polyploid cells. These findings further support that 10% serum concentration is not optimal for generating physiologically relevant IPEC-J2 monolayers.

Immunostaining for MUC2 revealed heterogeneous distribution patterns within the IPEC-J2 monolayers, consistent with mucin secretion from a subpopulation of cells rather than uniform expression across all cells (Fig. 6A2, B2, C2, and D2). MUC2 appeared as discrete, non-continuous patches overlying the monolayer, which was particularly evident in the 5PSAg condition (Fig. 6B2). This patchy distribution pattern resembles the in vivo situation where only goblet cells, not enterocytes, secrete MUC2. Co-labelling experiments using WGA, which binds to glycoproteins including mucins, alongside MUC2 antibody staining provided direct visualisation of mucin secretion (Fig. 6A1, B1, C1, and D1). These double-labelled images clearly demonstrated MUC2 secretion spreading from individual cells scattered throughout the monolayer (particularly visible in Fig. 6B1, seen also in Fig. 4B), strongly suggesting these are differentiated goblet cells. Immunostaining for TFF3 showed a more diffuse distribution pattern compared to MUC2 (Fig. 6A5, B5, C5, and D5). While TFF3 staining was detectable across all culture conditions, the signal appeared less concentrated and more dispersed throughout the monolayer. This diffuse pattern may reflect differences in the secretion kinetics or stability of TFF3 compared to MUC2, or could indicate that TFF3 is more readily released and spread across the epithelial surface.

The membrane-associated mucins MUC3 and MUC13 displayed distinct subcellular localisation patterns that differed from each other and from the secreted mucin MUC2. MUC3 immunostaining was predominantly detected at the periphery of cells, particularly in cultures subjected to agitation (Fig. 6B3 and D3). In contrast, MUC13 exhibited an unexpected intracellular localisation pattern, with staining concentrated in the perinuclear region rather than at the apical cell surface (Fig. 6A4, B4, C4, and D4). This intracellular accumulation was observed across all culture conditions. While MUC13 is expected to localise at the apical membrane in fully differentiated enterocytes, the perinuclear staining is observed here. This aberrant MUC13 localisation represents a notable difference between the IPEC-J2 model and native jejunal epithelium, where MUC13 is clearly present at the enterocyte surface (see Fig. 1K).

**Fig. 7:**
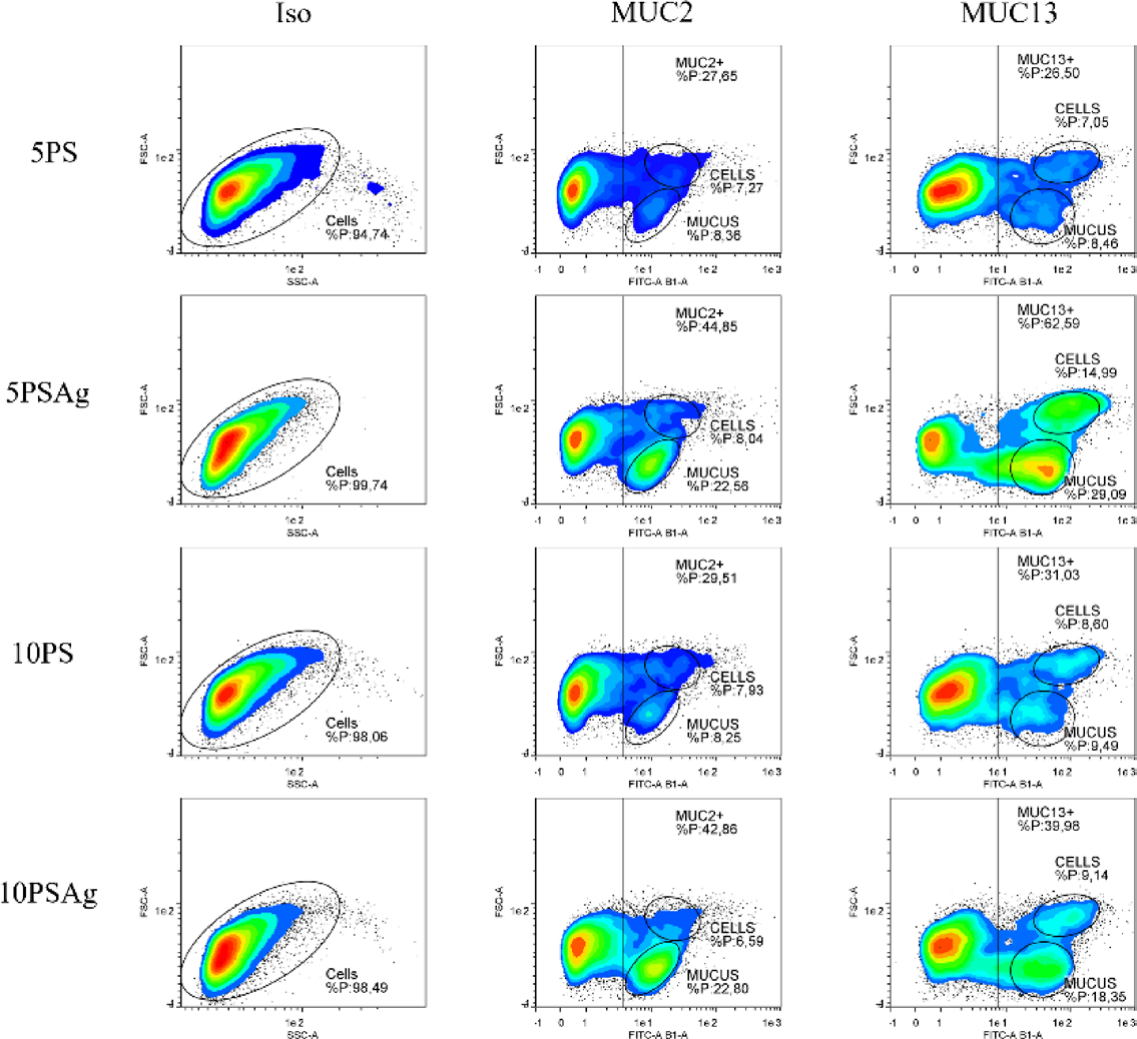
Representative flow cytometry dot plots quantifying MUC2 + and MUC13 + cell populations in IPEC-J2 cells cultured with 5% or 10% porcine serum with static (5PS or 10PS) or orbital agitation at 200 rpm (5PSAg or 10PSAg). Under optimal conditions (5PSAg), approximately 8% of cells express MUC2, matching the physiological goblet cell proportion observed in native piglet jejunum (~ 6%). Dual positive populations indicate cells co-expressing both membrane-bound (MUC13) and secreted (MUC2) mucins. Isotype control (Iso) establishes baseline fluorescence thresholds. Data representative of three independent experiments (*n* = 3).

In line with RT-qPCR and Western blotting results, flow cytometric analysis demonstrated that MUC2 and MUC13 expression increased by approximately 50% following cell agitation (Fig. 7). Detailed examination of the data revealed two distinguishable populations based on size characteristics, which may correspond to extracellular mucus produced by cultured cells and the mucin-producing cells themselves, respectively. Similarly to ex vivo data (Fig. 2), two populations were identified: the proportion of cells marked with the MUC2 was around 7.5%, while the other population, presumably mucus fragments, ranged from ~ 8% (5PS and 10PS) to ~ 22% (5PSAg and 10PSAg).

### Culture under air/liquid interface (ALI)

**Fig. 8:**
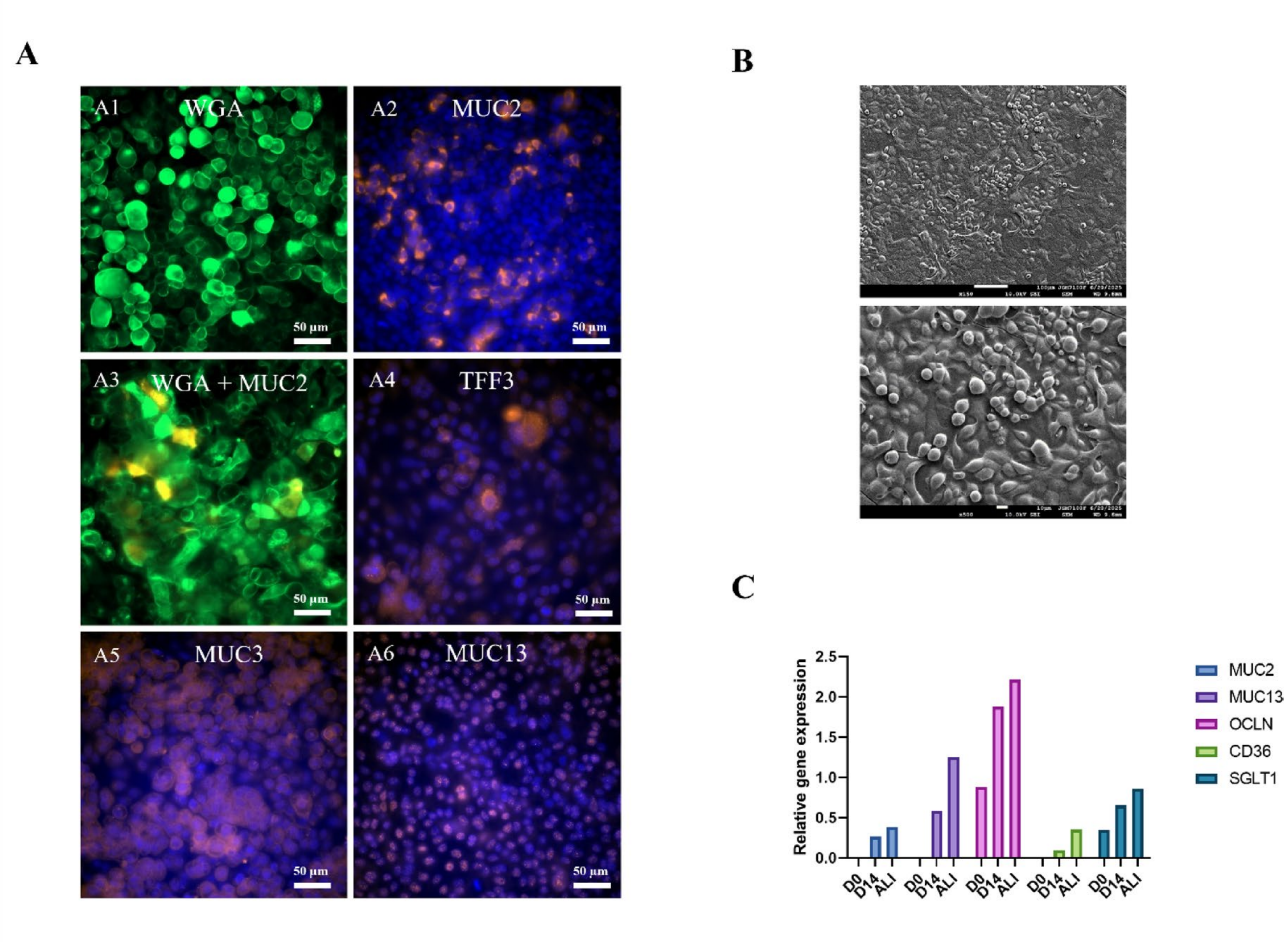
Immunofluorescence and electron microscopy of IPEC-J2 cells cultured under air/liquid interface conditions (ALI). WGA staining (green) shows extensive glycoconjugate networks, while labelling with MUC2, MUC3, MUC13, and TFF3 antibodies demonstrate robust mucin production potentially exceeding physiological jejunal levels. Nuclei were counterstained with DAPI (blue) (**A**). Scale bars: 50 μm. Scanning electron microscope showing the morphology of the IPEC-J2 monolayer under ALI condition (**B**). Longitudinal gene expression analysis comparing IPEC-J2 cultures maintained under 5PSAg (D0 and D14) or air-liquid interface (ALI) conditions (**C**). Enhanced expression of mucins (MUC2, MUC13) and tight junction protein (OCLN) genes with maintained transporter expression (CD36, SGLT1) demonstrated successful epithelial differentiation. Data represent mean ± SEM (*n* = 3). Images representative of three independent experiments.

Compared to the other four conditions, IPEC-J2 cells grown under ALI condition did not appear morphologically different (Fig. S1 and S2). Nevertheless, a viscous layer, presumably of mucus, was uniformly distributed across the surface under ALI condition (Fig. S2). SEM images depicted a monolayer similar in appearance to the 5PSAg IPEC-J2 with protuberances at regular interval within the sample (Fig. 8B). Immunochemistry images were comparable to the images obtained for the 5PSAg condition, however the mucins seemed more abundant, particularly MUC2 and MUC3 (Fig. 8A). This was confirmed by a preliminary experiment with a higher gene expression for ALI culture condition compared to 5PSAg for all markers studied (MUC2 and MUC13, tight junction proteins and nutrient receptors) (Fig. 8C).

## Discussion

Numerous studies have described the mucins secreted in the GIT of humans or rodents, particularly in the colon^6,22,26–29^. However, studies revealing the composition of mucus, including the mucins, in the pig jejunum remain scarce and non-exhaustive^20,30–32^. Examining the mucus at this site of the GIT is critical for understanding its interaction with dietary components and how it can influence nutrient hydrolysis and absorption^33–35^. The intestinal barrier function is ensured not only by the mucus layer but also by the intestinal epithelium itself^36^. This barrier prevents the passage of microorganisms and unwanted molecules through the epithelium while allowing nutrient absorption. Epithelial permeability is controlled by tight junction proteins such as occludin and ZO-1.

Our investigation of native piglet jejunal tissue confirmed the presence of MUC2, MUC13, and possibly MUC3. Non-specific staining using PAS and WGA revealed mucins both inside globular structures (goblet cells) and at the surface of the microvilli. TFF3 labelling, a marker for goblet cells, confirmed their size and location, although they appeared less numerous than when identified by non-specific stains or MUC2 labelling. Regarding membrane-bound mucins, MUC13 was detected and appeared evenly distributed throughout the jejunal tissue, specifically at the enterocyte surface, confirming its role in forming the glycocalyx in vivo. This observation aligns with existing literature describing MUC13 as a cell-surface mucin^7,37^. Conversely, MUC1 and MUC15 were not detected in the jejunal tissue. MUC3 was detected, but to a lesser extent than MUC13, and its presence was limited to only a few locations within the villi. These data provide a validated physiological reference, demonstrating that MUC13 is likely the main mucin constituent of the glycocalyx in the piglet jejunum.

IPEC-J2 cells have been well characterised for their permeability and tight junction structure, making them a good model to investigate the impact of microorganisms and pathogenic compounds on intestinal barrier function^38^. However, data on the composition of IPEC-J2 mucins are inconsistent, and not all mucins present in pig jejunum have been explored^20,30,39,40^. In this work, we tested different culture conditions since they can influence the growth and differentiation of IPEC-J2 cells and therefore their functional parameters, including mucin secretion^24,41^. We demonstrated by immunochemistry, RT-qPCR, and Western blot analyses that tight junction proteins (occludin and ZO-1) were present regardless of the conditions used. However, differences in TEER measurements suggested that agitation led to stronger barrier integrity compared to conditions without agitation. While TEER measurements confirmed the establishment of functional tight junctions, future studies could incorporate paracellular permeability assays using fluorescently labelled dextrans of various molecular weights (e.g., 4 kDa and 70 kDa FITC-dextran) to comprehensively assess barrier function. Such assays would reveal how the mucus layer produced under different culture conditions affects macromolecular diffusion and would provide functional validation of the barrier properties relevant to nutrient absorption and mucus-food compound interactions^14,42^.

Only a small proportion of cells present in IPEC-J2 cultures expressed MUC2, which seemed consistent with physiological conditions (see Fig. 1). Flow cytometry analysis estimated the quantity of MUC2-secreting cells in the 5PSAg sample at 8%. In humans, it has been reported that 6% of jejunal cells are goblet cells^11^, which is in agreement with our findings. MUC3 was clearly identified in 5PSAg and 10PSAg samples but not in all cells, and it did not form clusters as observed elsewhere^17^. MUC13 was detected as previously reported^43^, however not at the apical surface of the IPEC-J2 cells but inside the cells, around the nucleus. During its synthesis, MUC13 passes through the endoplasmic reticulum before travelling from the cytoplasm to the apical surface of the enterocytes^44^. Depending on the stage of differentiation and the culture conditions, it is possible that both the protein core, as suggested by our data, and the location of the mucins within the cell fluctuate. The chemistry of mucins is highly complex, and glycosylation could also modulate antibody binding to the mucin, compromising its identification^45^. Therefore, it is probable that MUC13 in vivo and in IPEC-J2 cells differed in their cellular location, their tertiary structure (despite similar molecular weight in vivo and in vitro), and/or their glycosylation patterns^44^.

 In vitro, culture conditions such as agitation and type of serum used (PS versus FBS) have a significant impact on cell characteristics, including mucus secretion. Indeed, previous studies reported that the serum type impacted monolayer integrity (trans- and paracellular resistance) and microvilli morphology, with IPEC-J2 cultured in PS being closer to pig tissue physiology^24^. Passage number is also likely to affect cell differentiation^15^. Therefore, attempts have been made to modulate IPEC-J2 culture conditions to evaluate their biological similarity to porcine tissue and their suitability for studying specific processes or mechanisms, such as cellular response to microorganisms and monolayer barrier function^24,25^. Using FBS and shorter growing periods resulted in IPEC-J2 cells that diverge from pig jejunum, hence the substitution of FBS with PS and a 14-day culture period^18,20,24,38,46^. Our results revealed that IPEC-J2 cultured in 10% PS without ITS and EGF produced abnormal cells, questioning their suitability as a jejunum model. Another important factor that is often overlooked is the movement applied to cells during their growth. The movements and contractions occurring in the GIT, such as peristalsis in the jejunum, stimulate mucus secretion^47^. Our solution to simulate these movements was to apply gentle agitation, as performed by another group on Caco-2/HT29-MTX-E12 co-cultures^48^, which succeeded in enhancing MUC2 expression.

The ALI culture condition is reported in the literature to enhance differentiation and more closely mimic physiological properties of intestinal cells^49,50^. Consistent with this, visual examination and immunochemistry suggested that mucin markers, particularly MUC2 and MUC3, were more abundant under ALI conditions compared to 5PSAg. However, the ALI condition generated a uniformly distributed and very dense mucus layer. This density made “extraction” of the cells challenging. Given that the small intestine in vivo is generally covered by a single, loosely adherent mucus layer, the dense layer produced under ALI conditions may not accurately represent the natural mucosal barrier of the jejunum. Therefore, while ALI promotes mucus-secreting cell (possibly goblet cells) differentiation, the resulting physical structure of the mucus layer may limit its suitability for interaction studies requiring a faithful representation of the jejunal environment.

The kinetic study carried out with 5PS shed light on the expression mechanisms of tight junction proteins and mucins. The digestive system of piglets undergoes major changes during development, in terms of enzyme secretion as well as the morphology of the intestinal epithelium and mucosal barrier^51,52^. Ideally, IPEC-J2 cells should be able to simulate some of these processes and to match specific physiological stages, in our case the piglet. We demonstrated that MUC2 expression increased during cell differentiation, confirming our previous discussion about culture time. The combination of conditions used in this work, PS and agitation, may explain why MUC2 was not expressed in certain studies^19,20^. Based on our findings, IPEC-J2 cells cultured under 5PSAg conditions (5% porcine serum with gentle agitation for 14 days) provide a suitable model for investigating dietary component interactions with intestinal mucus. Under these conditions, IPEC-J2 monolayers develop MUC2-secreting cells (presumably goblet cells) at physiologically relevant proportions, membrane-bound mucins, functional tight junctions, and differentiated enterocytes with apical microvilli. Despite limitations in MUC13 localisation, IPEC-J2 represents a useful tool for mechanistic studies of mucus-food compound interactions in the jejunum. The aberrant intracellular localisation of MUC13 observed in IPEC-J2 cells warrants further investigation. Future studies could employ co-localisation experiments with Golgi apparatus and endoplasmic reticulum markers to determine whether MUC13 is retained during biosynthesis and processing. Additionally, lectin-binding assays or mass spectrometry-based glycan profiling could reveal whether altered glycosylation patterns prevent proper trafficking to the apical membrane. Such investigations would clarify whether the MUC13 localisation observed here represents incomplete cellular differentiation or a fundamental limitation of the IPEC-J2 model.

Mucin secretion is triggered in vivo by various factors, including the presence of microorganisms, particularly bacteria that possess enzymes with mucolytic activity^53^. Diet can also play a role by influencing the bacterial population colonising the GIT or through direct interaction with the mucus layer. It is known that dietary fibres play an important role in this process, but other nutrients are also important, such as proteins^34,54,55^. Besides the class of molecules, their nature is also critical—oligosaccharides versus polysaccharides, or solubilised molecules versus intact cell walls. Diet can therefore impact mucus and mucin secretion in terms of both quantity and type.

For research involving the mucus layer, we therefore recommend growing IPEC-J2 cells in PS at a concentration of 5%, including ITS and EGF, with a low passage number (lower than 10 from reception from the supplier) and with agitation. Under these conditions, some of the cells within the IPEC-J2 culture should differentiate into mucus-secreting cells (goblet cells). While ALI culture conditions generated a thick mucus layer, this mucus may not be representative of that covering jejunal cells (too dense, more resembling colonic mucus).

## Conclusion

The IPEC-J2 cell line offers practical advantages for studying intestinal physiology when coupled with in vitro digestion systems. By exposing IPEC-J2 monolayers to digested food matrices, researchers can assess mucus-food compound interactions and nutrient absorption without surgical interventions such as cannulation or tissue sampling from live animals.

The combination of RT-qPCR, Western blotting, immunofluorescence, scanning electron microscopy, and flow cytometry revealed comprehensive mucin characterisation at gene, protein, and cellular levels. Flow cytometry quantified that approximately 8% of IPEC-J2 cells secrete MUC2 under optimal conditions, closely matching the proportion of goblet cells in native jejunum.

However, important differences exist between IPEC-J2 cultures and native jejunal tissue. MUC13 showed aberrant intracellular localisation rather than proper apical membrane positioning observed in vivo, and the proportion of mucus-secreting cells varied with culture conditions. Single-cell RNA sequencing would provide comprehensive insight into cellular heterogeneity within IPEC-J2 cultures and clarify whether different culture conditions generate distinct cell subpopulations (enterocytes, goblet cells, potentially others) or a continuum of differentiation states. Multi-marker flow cytometry incorporating TFF3, cytokeratin 20 (KRT20), and brush border enzymes (sucrase-isomaltase) would enable more precise quantification of cell populations. Fluorescence-activated cell sorting followed by targeted qPCR or RNA-seq would definitively confirm the presence of distinct goblet cell and enterocyte populations and their respective proportions under optimal culture conditions.

Finally, further studies should focus on the physico-chemical properties of the mucus layer produced under different culture conditions of IPEC-J2 such as particle tracking to assess micro-rheology.

## Methods

### Intestinal tissue collection

Jejunal tissues were collected in 4-to-6-day-old, suckling piglets (Pietrain × (Large White × Landrace), males and females, *n* = 6) that came from the herd of the INRAE UE3P experimental facility (INRAE, Saint-Gilles, France, 10.15454/1.5573932732039927E12; Approval number D35-275-32; protocol P23-009). All procedures used in this study were in compliance with the ethical standards of the European Community and French regulations (Directive 2010/63/EU, and French Law 2013–118, February, 2013) and in accordance with ARRIVE guidelines (https://arrivequidelines.org). Following slaughter, 4 cm sections of the jejunum were collected (at 1 m from the pylorus), immediately fixed in 4% formaldehyde and dehydrated before being embedded in paraffin for histochemical analyses. About 1 g of jejunum was also collected, cut into small pieces, immediately frozen in liquid nitrogen and stored at -80 °C until molecular biology analyses.

### Cell culture and transepithelial electrical resistance (TEER) measurement

The porcine jejunal intestinal cell line IPEC-J2 was obtained from DSMZ (ACC-701, Braunschweig, Germany). The IPEC-J2, passages 3 to 6, were grown in 75 cm^2^ flasks containing Dulbecco’s Modified Eagle Medium/Ham’s F-12 (DMEM/F12, Gibco, reference 31331028) supplemented with 10% porcine serum (PS) containing 1% penicillin-streptomycin (Gibco, reference 15140122) as previously described^14^. At 80% confluence, cells were seeded on transwell polyester membrane inserts (0.4 μm pore size, 1.1 cm^2^ surface area), placed in 12-well plates at a density of 1 × 10^5^ cells per cm^2^ (Sarstedt, reference 2582608), and in DMEM/F12 medium supplemented with either 5% PS, 1% penicillin-streptomycin, 1% Insulin Transferrin Selenium (ITG, Gibco, reference 41400045), and 10 µg/mL Epithelial Growth Factor (EGF, Merck, reference SRP3027-500UG); or 10% PS and 1% penicillin-streptomycin. For these two conditions, 5 or 10% PS, the cells were culture without (5PS and 10PS) or with orbital agitation (5PSAg and 10PSAg) at 200 rpm (Microplate Mixer, N2400-8040, StarLab, Hamburg, Germany). The cells were grown for 14 days at 37 °C under 95% humidity (95%) and 5% CO_2_ in conditions based on previous studies^24,25,48^. The medium was changed every two to three days and the transepithelial electrical resistance (TEER) measured on day 4, 7, 11 and 14 with an Epithelial Voltohmmeter (EVOM3, Friedberg, Germany).

TEER values, in Ω × cm^2^, were calculated as follows:

1 $$TEER{\text{ }} = {\text{ }}(R - R0){\text{ }} \times A$$

where R is the measured resistance (Ω), R0 is the blank resistance (insert without cells), and A is the inset effective surface (1.1 cm^2^).

For the air-liquid interface (ALI) culture condition, the cells were cultured in 12-well plates with inserts in 5% PS media as described above (without agitation) for 7 days. The apical media was then removed and the cells were left to differentiate for a further 7 days (14 days in total), changing the basal medium every two to three days.

Three independent cell culture experiments were performed (*n* = 3 biological replicates). Each experiment included technical triplicates. During the culture, the cells were visualised using an Axio Zeiss Vert. A1 microscope with a 20x objective lens coupled with an Axio Cam MRC camera (Zeiss, Marly-le Roi, France).

### Immunochemistry

For jejunum tissues (*n* = 6 piglets), the sections were dehydrated before being embedded in paraffin. Three independent sections of 5 μm thickness were subsequently cut per animal using a microtome and mounted on glass slides. The sections were then deparaffinised in xylene and rehydrated through a graded ethanol series before proceeding to the staining/immunolabeling steps. The obtained samples were either stained with Periodic acid–Schiff (PAS) or labelled with wheat germ agglutinin (WGA) and then observed under a light microscope (Zeiss, AXIO Imager M2, France).

After 14 days of differentiation in the different conditions, the IPEC-J2 were fixed with methanol, washed twice in phosphate-buffered saline (PBS) solution, permeabilized with Triton X100 and washed twice again with PBS. The monolayers were then incubated with either WGA staining, MUC2 or Zonula occludens 1 (ZO-1) antibodies as previously described^14^. For MUC1, MUC3, MUC13, MUC15 and TFF3 antibodies, the procedure was similar to the one used for MUC2 (see Tables S1 and S2 for details of the antibodies). The samples were mounted on microscopy slides with 15 µL of ProLongTM Gold Antifade Mountant with DNA Stain 4’,6-diamidino-2-phenylindole (DAPI) to stain the nuclei and vizualised with a Zeiss Axio Imager M2 microscope. Additionally, the MUC2 and WGA + MUC2 5PSAg samples were observed with a Celldiscoverer 7 confocal laser scanning microscope (ZEISS). Images were captured using a 50x objective lens. The samples were excited using an argon laser at 353 nm for DAPI, 488 nm for WGA, and 545 nm for MUC2. The fluorescence emitted by the samples was detected at 400 to 500 nm (DAPI), 495 to 545 nm (WGA) and 530 to 617 nm (MUC2). The images were processed with the software Qupath (https://qupath.github.io).

### RT-qPCR analyses

Extraction of RNAs and the quantification of their expression levels was performed as previously described^56^. Total RNAs were extracted from cells or intestinal tissue using commercial kits (NucleoSpin RNA XS kit or Nucleospin RNA respectively, Macherey-Nagel, Düren, Germany) according to the manufacturer’s instructions. DNA from cells RNA preparations was removed using the DNA-free kit (Life technologies, Carlsbad, USA). The quality and amount of extracted RNA were estimated using a microvolume spectrophotometer DS-11 (DeNovix, Wilmington, USA). The integrity of extracted total RNAs was assessed using the RNA 6 000 Nano kit (Agilent Technologies France, Massy, France) with the Agilent 2100 Bioanalyzer (Agilent Technologies). First-strand cDNA synthesis was performed using SuperScript IV Vilo with EZDNase kit according to the manufacturer’s instructions (Thermo Fischer Scientific, Carlsbad, USA). Primers and probes were designed from porcine sequences available in Ensembl or NCBI databases using Primer Express^®^ v3.0 software (Thermo Fischer Scientific). Detailed information on the sequences (probes, forward and reverse primers) is provided in Table S3.

Expression levels of the genes were quantified using the SybrGreen technology. For each gene, the amplification efficiency (E) of qPCR reaction was determined using calibration curves generated with decreasing concentrations of cDNA from pooled samples (obtained from 10 to 0.625 ng RNA). All reactions were carried out on a StepOnePlus™ Applied Biosystems real-time PCR system (Thermo Fischer Scientific). Amplification reaction was performed in duplicate in 12.5 µL with 2.5 ng of reverse-transcribed RNA and amplification conditions were as follows: 2 min at 50 °C, 20 s at 95 °C followed by 40 cycles of 3 s at 95 °C, 30 s at 60 °C. Specificity of the amplification products was checked by dissociation curve analysis. Tyrosine 3-Monooxygenase/Tryptophan 5-Monooxygenase Activation Protein Zeta (YWHAZ), beta-2 microglobuline (B2M) and Ribosomal Protein L4 (RPL4) were identified as the most stable housekeeping genes in the intestinal tissue and Actin Beta (ACTB) and Pseudogene Similar To Part Of Heat Shock 90kD Protein 1, Beta (HSPCB) in cells, respectively, and were used to calculate the normalization factor (NF)^57^. For all examined genes, the normalised expression level N was calculated according to the formula developed by Pfaffl^58^:

$$\:N=\frac{{E}^{-\varDelta\:Cq\:(sample-calibrator)}}{NF}$$

where E is calculated from the amplification efficiency, Cq is the quantification cycle, and calibrator is a pool of all samples. For all studied genes, E was between 1.88 and 2.01.

### Protein extraction and Western blotting

Proteins were extracted from cell cultures using radioimmunoprecipitation buffer, quantified with the bicinchoninic acid assay kit (23227, Thermo Fisher, Illkirch, France) and analysed by Western blotting. The amount of loaded protein was to 10 µg per sample. Enhanced chemiluminescence (ECL) signal was digitalised using the ImageQuant LAS4000 Imager digital system (GE Healthcare, Velizy-Villacoublay, France) and quantified with the ImageQuant TL software (GE Healthcare). The antibodies used are described in Tables S1 and S2.

### Scanning electron microscopy

After 14 days of culture, the IPEC-J2 were rinsed in cacodylate buffer before being fixed with 2.5% glutaraldehyde, and postfixed in 1% osmium tetroxide. The samples were dehydrated in a graded ethanol series (50, 70, 80, 90 and 100%) and dried in a Leica EM CPD300 critical point dryer. The samples were then mounted into a metal support using double-sided carbon tape. A metallisation by cathodic sputtering was carried out with gold palladium for 40 s in a Leica EM ACE200. The samples were viewed with a scanning electron microscope (JEOL JSM 7100 F, Zaventem, Belgium).

### Flow cytometry

About 5 g of jejunal tissue (see above “Intestinal tissue collection”) were collected under sterile conditions and transported in ice-cold DMEM supplemented with 10% foetal bovine serum (FBS) and antibiotics. Tissue segments were opened longitudinally, rinsed with PBS to remove luminal contents, and cut into 2–3 mm² pieces. Tissue pieces were first incubated in 5 mM EDTA solution at 37 °C for 30 min with gentle agitation to chelate divalent cations and facilitate epithelial cell release. Following PBS washes, tissues were subjected to enzymatic digestion using a cocktail containing 1 mg/mL collagenase Type IV, 0.1 mg/mL dispase II, and 0.01 mg/mL DNase I in DMEM with 2% FBS at 37 °C for 45–60 min. The digested tissue was mechanically disrupted and sequentially filtered through 70 μm and 40 μm cell strainers to obtain single-cell suspensions. Cell pellets were treated with ACK lysis buffer for 3 min to eliminate red blood cells, washed twice with PBS, and assessed for viability using trypan blue exclusion.

Isolated cells or IPEC-J2 cells were incubated in MACS buffer with the MUC2 or MUC13 antibody (see Tables S1 and S2) for 20 min at 4 °C in the dark, washed and re-suspended in autoMACS rinsing buffer with 2% MACS bovine serum albumin for cell phenotyping. Flow cytometry was performed using a MACSQuant Analyzer 10 cytometer (Miltenyi Biotec, Paris, France). Pre-cytometry viability assessment included (i) cell viability evaluated using the TC20 Automated Cell Counter (Bio-Rad) immediately before flow cytometry analysis, and only samples with viability > 95% were processed for cytometry; and (ii) morphological gating based on FSC/SSC to exclude debris and doublets. The gating strategy applied consisted in, first, identifying the intact cells based on forward scatter area (FSC-A) versus side scatter area (SSC-A) to exclude debris and non-cellular events. Single cells were then selected using FSC-A versus FSC-height (FSC-H) to eliminate doublets and cell aggregates. Isotype control antibodies (rabbit IgG matched to the primary antibody) were used as negative controls to establish fluorescence thresholds. MUC2 and MUC13 positivity was determined within the gated cell population. A minimum of 10,000 events were acquired per sample. Data were analysed using MACSQuantify analysis software (Miltenyi Biotec, Paris, France), and the percentage of positive cells was calculated from the single-cell gate.

### Statistical analysis

The data were analysed using R studio version 4.1.2. All data were expressed as means of triplicates ± SEM. For kinetic analysis, one-way ANOVA was used with time point as factor. For the other analyses, two-way ANOVA, followed by Tukey’s post-hoc test, was carried out including culture condition (4 levels: 5PS, 5PSAg, 10PS, 10PSAg) and time (4 levels: days 4, 7, 11, 14) as main effects on TEER measurements, or the effects of serum concentration (5% or 10%) and agitation (static or agitated) and their interaction as main effects for gene expression. Before ANOVA, normality was assessed using Shapiro-Wilk tests, and homogeneity of variance was evaluated using Levene’s test. All data met the assumptions for parametric analysis (*P* > 0.05 for all tests). The significance level was set at *P* < 0.05 and *t* indicated a statistical trend (0.05 < *P* < 0.10).

This work was supported by INRAE and the Agence Nationale de la Recherche (ANR, grant number ANR-25-CE20-0228).

## Supplementary Information

Below is the link to the electronic supplementary material.

Supplementary Material 1

## Data Availability

All data supporting the findings of this study are available within the paper and its Supplementary Information.
