# Supplementary material for: IPEC-J2 as a cellular model for studying intestinal mucus
Source: Sci Rep. 2025 Dec 9;16:2338. doi: 10.1038/s41598-025-32027-5 (PMC12816010; doi:10.1038/s41598-025-32027-5)

Supplementary information

Table S1: Information on primary antibodies used in the study

| **Target** | **Provider** | **Reference** | **Clonality & clone** | **Fc domain** | **Dilution** |
| --- | --- | --- | --- | --- | --- |
| MUC1 | Antibodies Online | ABIN6736343 | Polyclonal | Rabbit IgG | 1:200 |
| MUC2 | Invitrogen | PA5-119292 | Polyclonal | Rabbit IgG | 1:200 |
| MUC3* | Abcam | Ab270273 | Monoclonal, 2992R | Rabbit IgG | 1:200 |
| MUC13* | Abcam | Ab235450 | Monoclonal, EPR21901 | Rabbit IgG | 1:200 |
| MUC15 | Antibodies Online | ABIN6741755 | Polyclonal | Rabbit IgG | 1:200 |
| TTF3* | Abcam | Ab108599 | Monoclonal, EPR3974 | Rabbit IgG | 1:200 |
| ZO-1 | Invitrogen | 339194 | Monoclonal, Alexa Fluor 594 conjugate | Mouse IgG | 1:100 |
| OCLN | Abcam | Ab216327 | Monoclonal, EPR20992 | Rabbit IgG | 1:100 |

*Recombinant antibodies

Table S2: Information on secondary antibodies used in the study

| **Species reactivity** | **Provider** | **Reference** | **Conjugate** | **Host** | **Dilution** |
| --- | --- | --- | --- | --- | --- |
| Rabbit IgG | Cell Signaling | #4413 | Alexa Fluor 555 | Goat | 1:400 |

Table S3: Primers used for the RT-qPCR analyses

| **Gene symbol** | **Accession number^1^** | **Primer sequence (5’ 3’)** |
| --- | --- | --- |
| MUC2 | XM_021082584 | F : ggctgctcattgagaggagt  R : atgttcccgaactccaagg |
| MUC13 | NM_001105293 | F: GAGACTGGCTTTAGCAACCT  R: AGTCTATCAAACCCTCACAC |
| OCLN | NM_001163647 | F: CATGGCTGCCTTCTGCTTCATTGC  R: ACCATCACACCCAGGATAGCACTCA |
| ZO1 | XM_003121672 | F : AGGCGATGTTGTATTGAAGATAAATG  R : TTTTTGCATCCGTCAATGACA |
| CD36 | NM_001044622 | F: GCACAGAAAAAGTTGTCTCCAAAAAT  R: ATGTACACAGGTTTTCCTTCTTTGC |
| SGLT1 | NM_001164021 | F : cccaaatcagagcattccattca  R : aagtatggtgtggtggccggtt |
| ACTB^2^ | ENSSSCT00000042531 | F : TCTGGCACCACACCTTCT  R : TGATCTGGGTCATCTTCTCAC |
| B2M^2^ | NM_213978 | F: GCCATTCCAGTCAGGTTCTACAC  R: TCCAATAGAATTCCACACCATGAA |
| HSPCB^2^ | NM_001244433 | F : GGCAGAAGACAAGGAGAAC  R : CAGACTGGGAGGTATGGTAG |
| RPL4^2^ | ENSSSCT00000087662 | F : AGGAGGCTGTTCTGCTTCTG  R : TCCAGGGATGTTTCTGAAGG |
| YWHAZ^2^ | XM_001927228 | F: ATGCAACCAACACATCCTATC  R: GCATTATTAGCGTGCTGTCTT |

^1^Accession number in the National Center for Biotechnology Information (NCBI) and Ensembl project databasis for pig sequences. F and R indicated forward and reverse primers, respectively

^2^ Gene used as reference for normalization

Abbreviations: MUC2: Mucin 2; MUC13: Mucin 13; OCLN: Tight junction protein occludin; ZO1: Tight junction protein Zona Occludens 1; CD36: Fatty Acid Translocase; SGLT1: Solute Carrier Family 5 Member 1; ACTB: actin beta; B2M: beta-2 microglobuline; HSPCB: heat shock protein 90 alpha family class B member 1; RPL4: Ribosomal Protein L4; YWHAZ: Tyrosine 3-Monooxygenase/Tryptophan 5-Monooxygenase Activation Protein Zeta


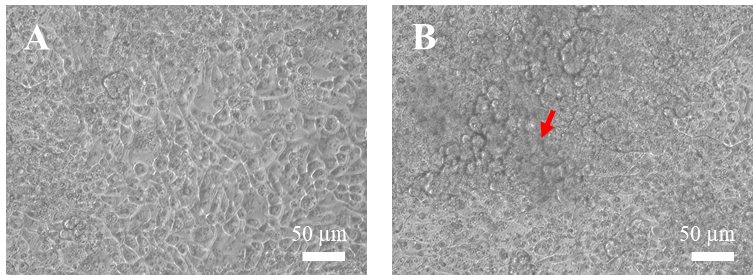


**Figure S1.** Typical images of IPEC-J2 monolayer after 14 days of culture for A) 5PS, 5PSAg, 10PS and 10PSAg, and B) 5PSAg with a viscous layer above the cells (red arrow) probably corresponding to mucus.


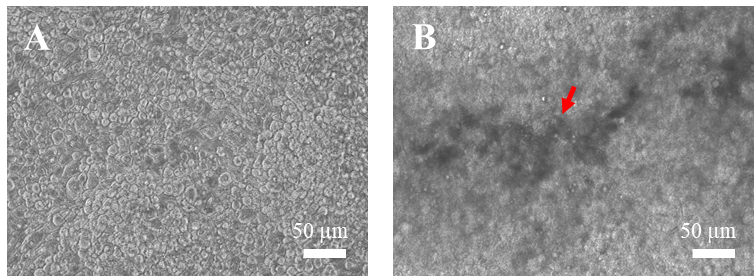


**Figure S2.** Typical images of IPEC-J2 monolayer after 14 days of culture under ALI condition. Note in B) the viscous layer (red arrow) that is visible above the cells (probably mucus).


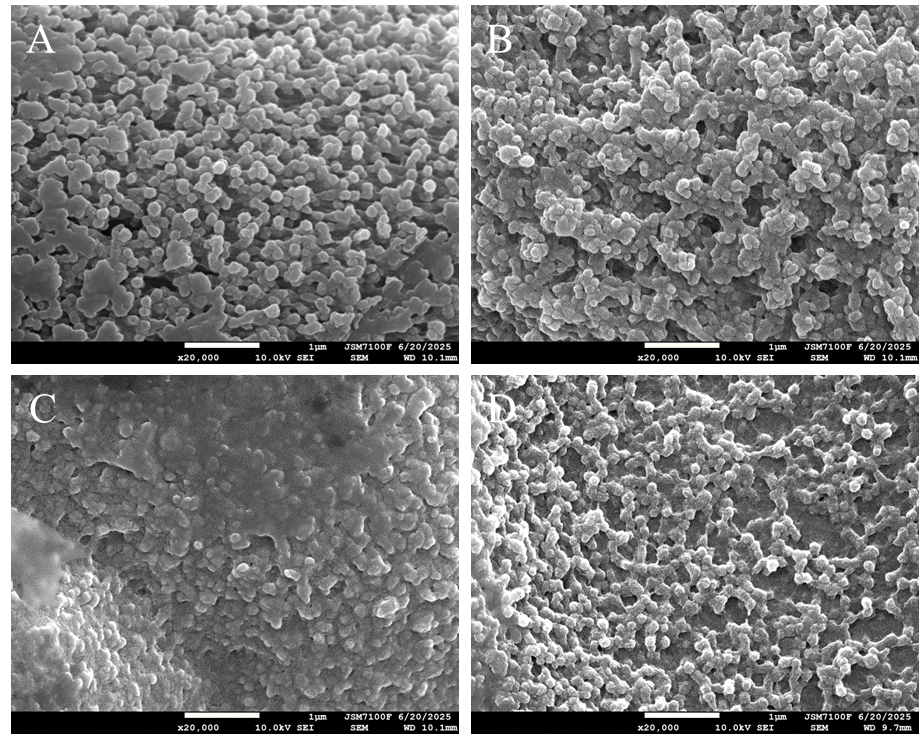


**Figure S3.** SEM images showing apical microvilli for 5PS (A), 5PSAg (B), 10PS (C), and 10PSAg (D).

**Full length blots**

**Figure S4** (Figure 2 in the manuscript)

MUC2


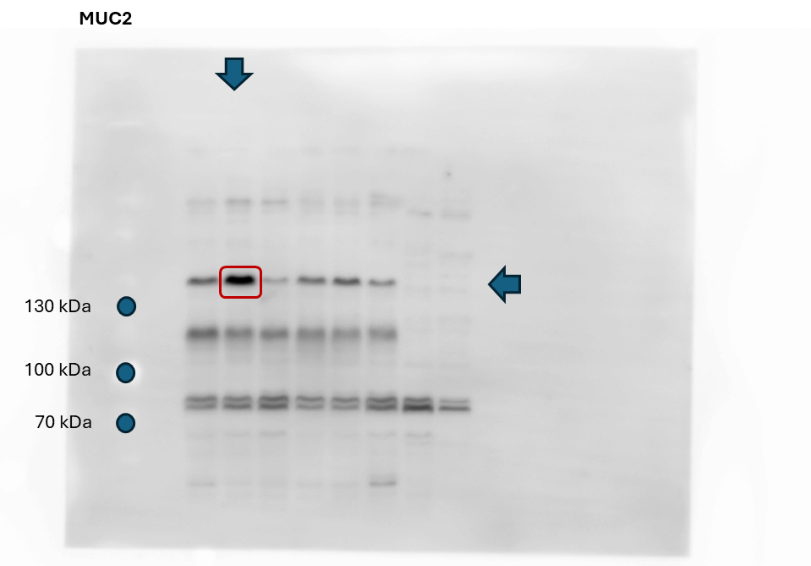


MUC13


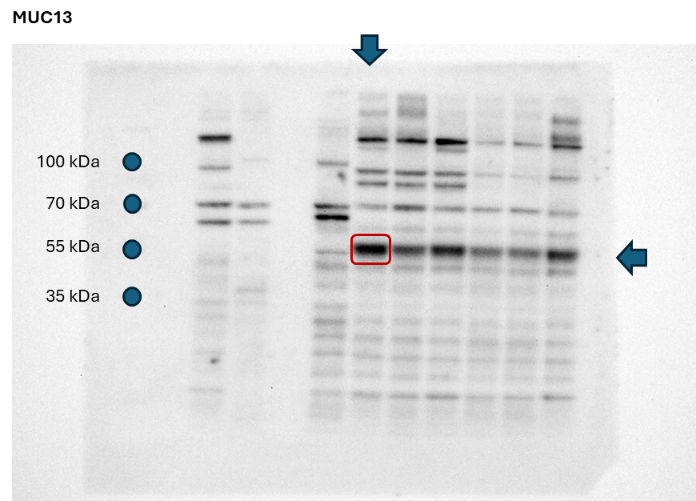


TFF3


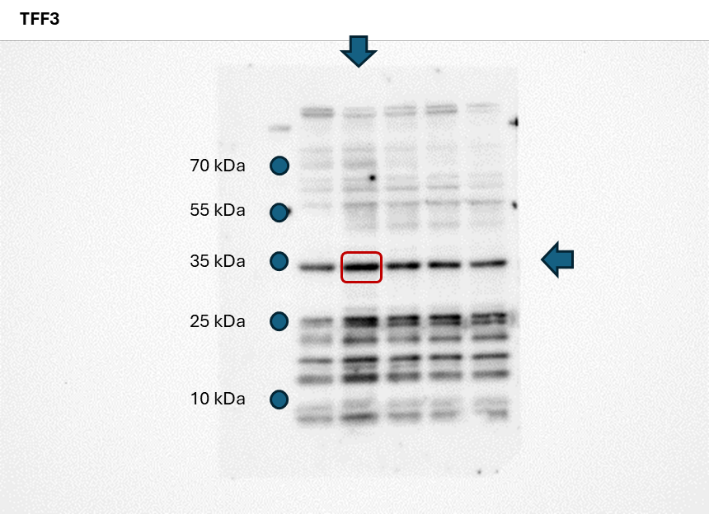


OCLN


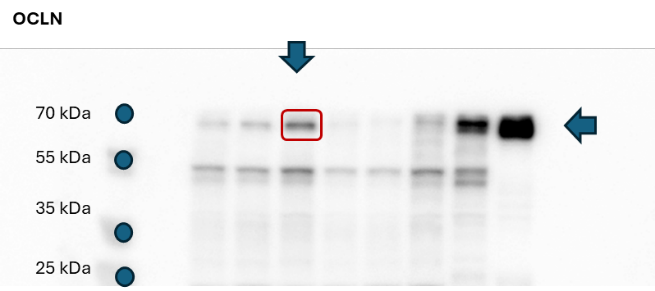


ZO-1


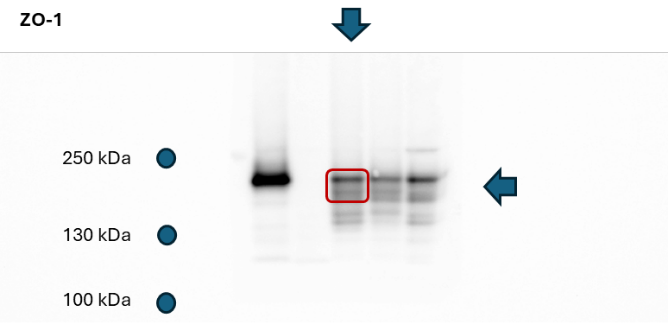


**Figure S5** (Figure 3 in the manuscript)

MUC2


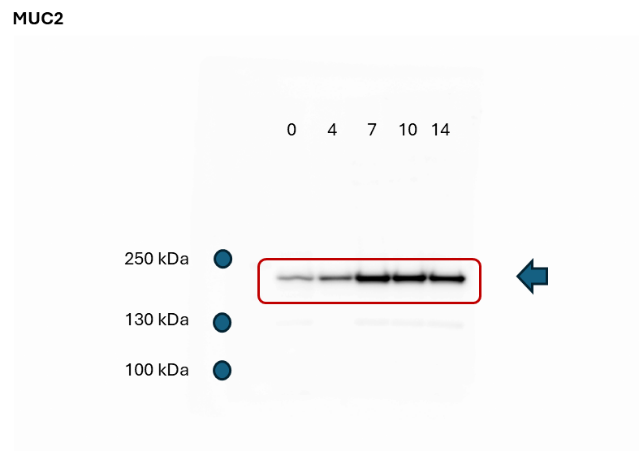


MUC13


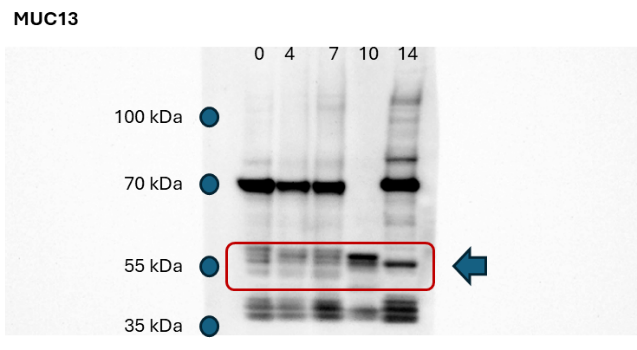


TFF3


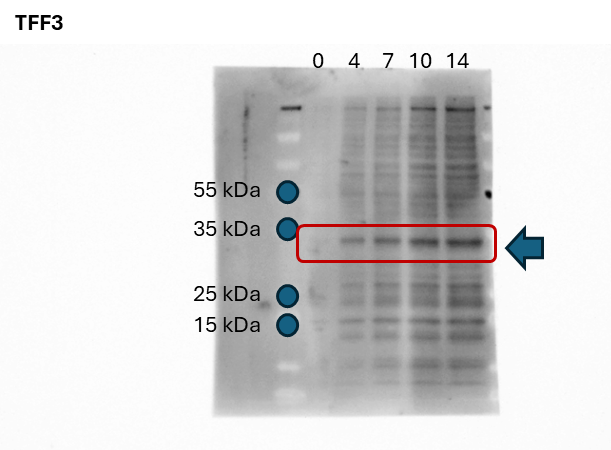


OCLN


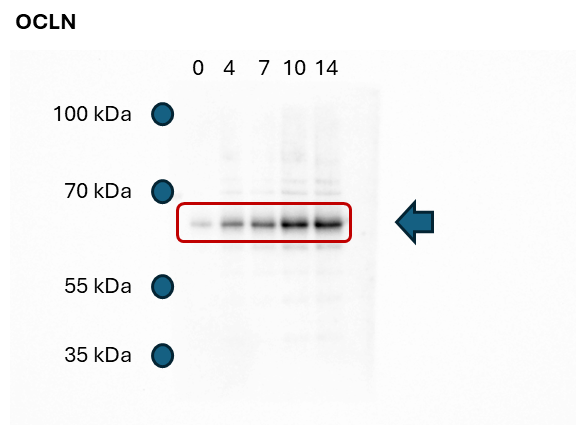


ZO-1


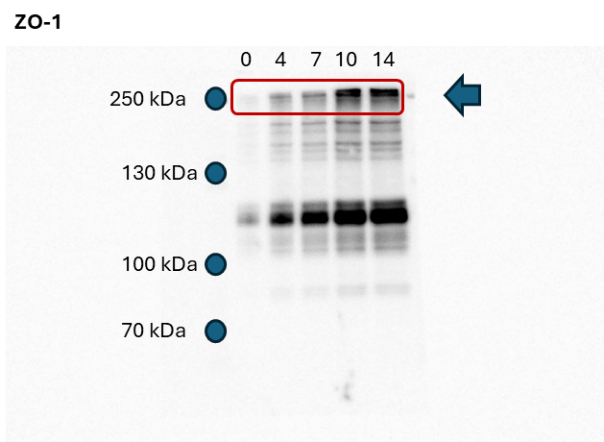


ACTIN


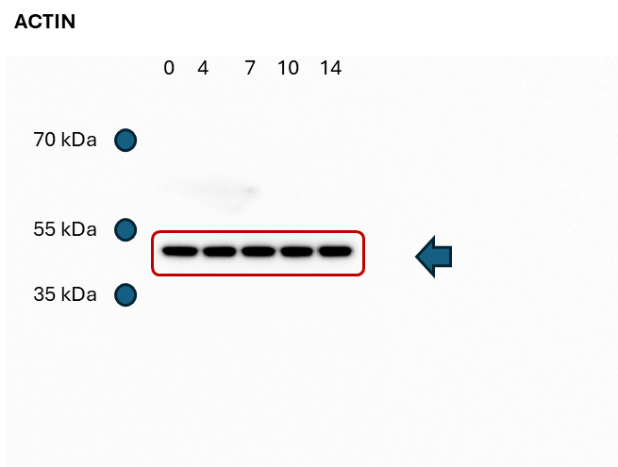


**Figure S6** (Figure 5 in the manuscript)

MUC2


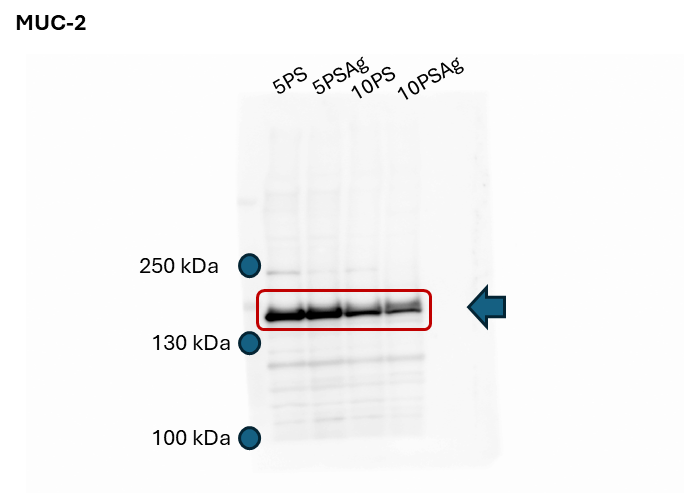


MUC13


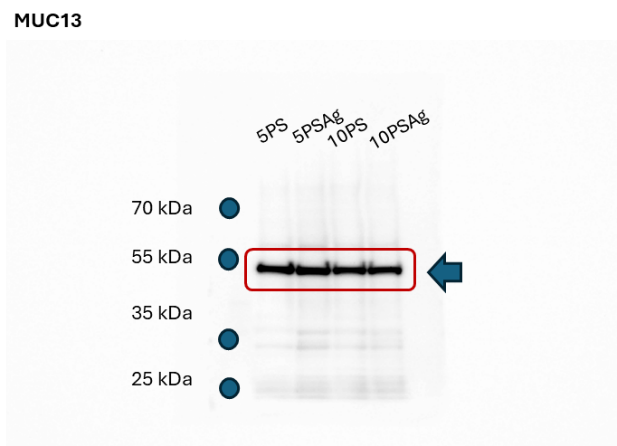


TFF3


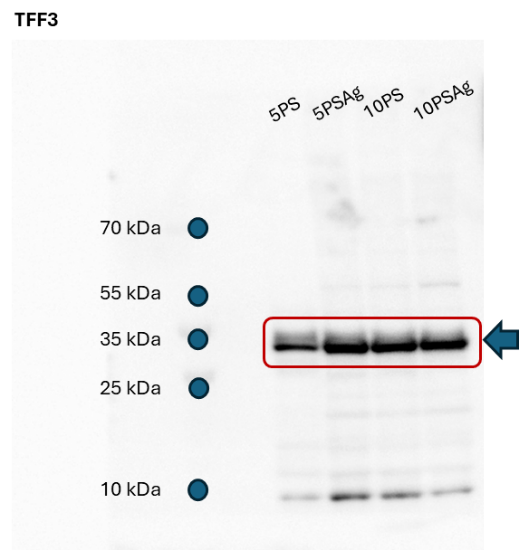


ZO-1


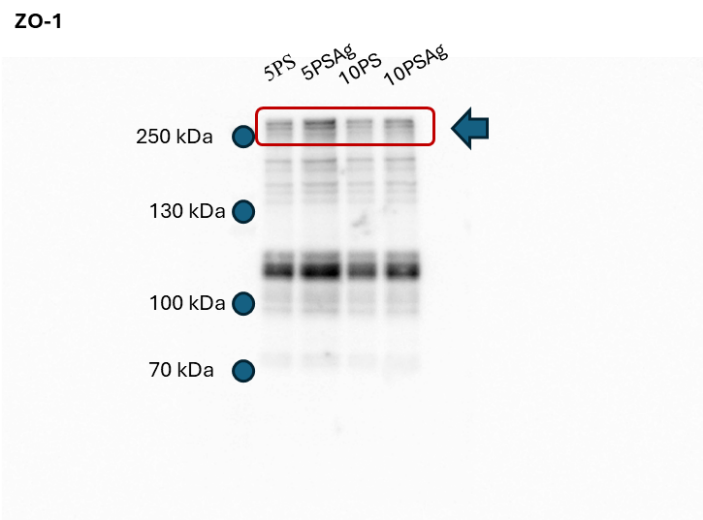


OCLN


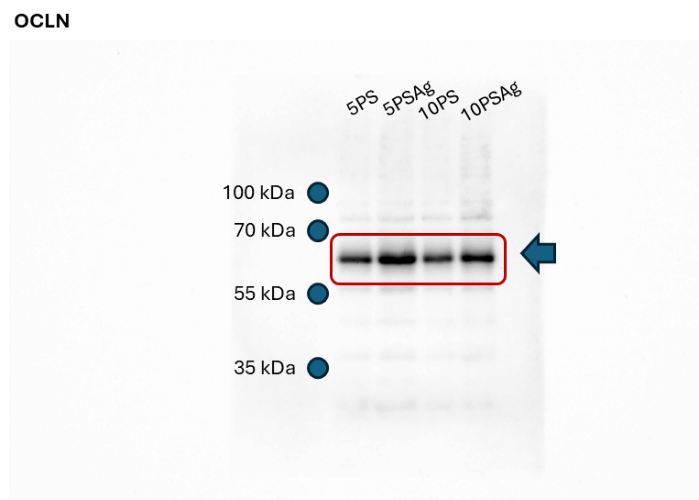


ACTIN


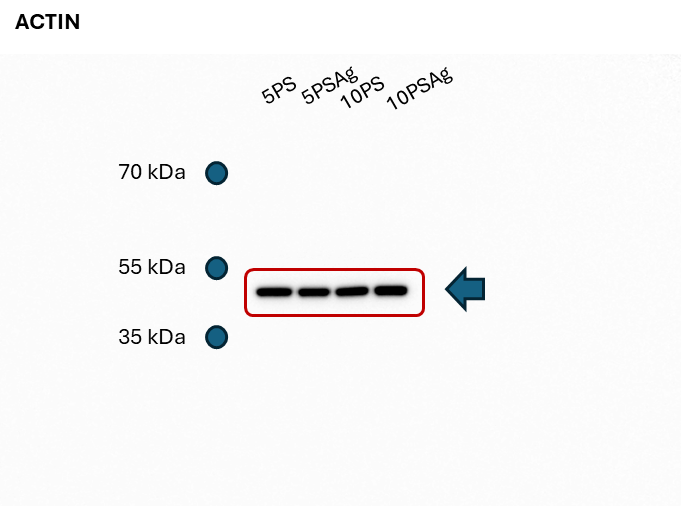

Supplement: Supplementary file 1 — Supplementary Material 1 [file 41598_2025_32027_MOESM1_ESM.docx]
